# Distressing Visual Hallucinations after Treatment with Trazodone

**DOI:** 10.1155/2017/6136914

**Published:** 2017-06-18

**Authors:** Gustavo Santos, Ana Maria Moreira

**Affiliations:** Hospital de Magalhães Lemos, Porto, Portugal

## Abstract

Trazodone, a second-generation atypical antidepressant, is increasingly being used off-label, in the treatment of insomnia. Although generally well tolerated, trazodone treatment can be associated with some complications. We describe a case of a 60-year-old man who received trazodone for primary insomnia. He returned, to the emergency department, two days later with distressing visual hallucinations, which prompted inpatient treatment. Trazodone was discontinued, leading to a complete resolution of his visual hallucinations, and he was treated with mirtazapine for 6 months. There has been no relapse in a follow-up period of two years. Patients presenting with visual hallucinations without significant psychiatry history can be a challenging situation. We highlight the importance of careful anamnesis with an accurate medication history. Given the widespread use of trazodone, clinicians should be aware of this possible side effect.

## 1. Introduction

Trazodone is a commonly prescribed atypical antidepressant with hypnotic properties and it is used as a short-term treatment strategy to improve sleep in a number of psychiatric disorders [1, 2]. It is generally well tolerated with a favorable side effect profile [3, 4], but a recent review suggested that more studies are necessary to investigate possible new therapeutic indications and to scientifically demonstrate the benefit ratio, for the many conditions for which trazodone is used [5].

Hallucinations are sensory perceptions that occur in the absence of an external stimulus, in any sensory modality. Visual Hallucinations are more common in acute organic states with clouding of consciousness and in patients with neurodegenerative disorders. We report a case of trazodone-induced visual hallucinations that, to our knowledge, is the first described in the literature.

## 2. Case Presentation

A 60-year-old widower presented to the emergency department (ED) with a two-day history of visual hallucinations and was promptly referred to psychiatry assessment. He had hypertension and his daily medication included Amlodipine 5 mg and Losartan 50 mg. Though he had history of alcohol abuse, he had been consistently abstinent for the last ten years. The patient denied current and past use of illicit drugs, including Lysergic Acid Diethylamide (LSD) and other hallucinogens drugs.

Three days ago, he went to his GP, complaining about having difficulty falling asleep and frequent waking during the last week. He denied any associated snoring, abnormal movements, or leg twitching. In the absence of any emotional and behavioral problem, he has been diagnosed with primary insomnia, and he has been prescribed with trazodone 100 mg. Soon after he started the treatment, his insomnia improved slightly and he began to describe complex visual hallucinations. He was able to clearly see dead familiars, who were standing in front of him, waving at him, when he was sleeping, or when he was performing his daily activities. All of them have passed away several years ago. He knew that these visions were not real but he was frightened and concerned he could be losing his mind. These visions lasted only few minutes and tended to occur more often during the evening and were not related to the sleep initiation or termination. The experiences occurred in clear consciousness and he was able to continue whatever he was doing during the hallucinations.

There was no report of perceived stressor preceding current episode. The patient had no associated medical comorbidities such as coronary heart disease, obstructive airway disease, or endocrine abnormalities. The patient had no history of head injury, migraines, central nervous system infection, and significant medical or psychiatric illness, including mood or psychotic disorder. His family history for psychiatric disorders was unremarkable, as well.

On examination, he was apyrexial, with a blood pressure of 111/83 mmHg, a pulse of 88 beats per minute, and a respiratory rate of 18 breaths per minute. Confrontational visual field test did not show any visual impairment. On mental state examination, the patient was oriented both in time and space and described vivid and complex visual hallucinations. He did not develop a complex delusional system to explain this phenomenon and he denied hallucinations to sensory modalities other than vision. The remaining physical and neurological examination was normal. The Mini Mental State Examination (MMSE) was negative (27/30) for cognitive dysfunction.

The patient was then admitted to the psychiatry department for etiological study and treatment.

The following investigations were performed. Drug urine screening was negative to illicit substances. CBC (diff), electrolytes, thyroid function test, and liver enzymes were normal, along with a negative blood alcohol level. A head CT scan excluded acute ischemic or hemorrhagic lesions but revealed mild ischemic leukoencephalopathy and a stable small chronic infarct in the right basal ganglia. Neurology consultation was done by a neurologist with expertise on neuropsychiatry and was unremarkable.

## 3. Differential Diagnosis

There are many etiologies for visual hallucinations; therefore many differential diagnoses were hypothesized.

Delirium is a syndrome that involves an acute disturbance of consciousness and a diminished ability to sustain attention with multiple etiologies. This patient was known to have used alcohol in a dependent fashion, in the past, and visual hallucinations are relatively common in drug withdrawal syndromes. However, the patient had a negative alcohol blood level and negative urine drug test. Furthermore, visual hallucinations occurred in clear consciousness with sustained attention and were vividly recalled, both circumstances arguing against a delirium syndrome.

Visual hallucinations are also found in neurodegenerative disorders [6, 7]. They occur commonly in Parkinson's disease and dementia with Lewy bodies, and less frequently in other neurodegenerative causes of parkinsonism, such as multiple system atrophy, progressive supranuclear palsy, and corticobasal degeneration syndrome [8]. However, the preservation of cognitive function and the absence of any parkinsonian feature and functional impairment make the diagnosis of dementia unlikely [9].

Visual hallucinations are also present in Charles Bonnet Syndrome, which is a neurological disease characterized by recurrent visual hallucinations usually following visual loss [10]. It typically occurs in older persons and it might be the clinical hallmark of the deafferentation of the visual cortex [11]. They may also be present in a rare condition, Anton's syndrome, in which patients with cortical blindness deny that they have visual loss [12]. This patient had no visual impairment and his neurological examination was unremarkable.

Sensory phenomena such as hallucinations have been described within the complex clinical presentation of epilepsy, as epileptic discharges that occur in the primary visual cortex or visual association may produce sensory seizures. Albeit rare, a seizure with only subjective visual hallucination can occur in isolation [13]. Usually it is stereotyped and lasting only for seconds [13]. Moreover, a recent review showed that current use of antidepressants was associated with a twofold increased risk of first-time seizures compared with nonuse [14]. Nevertheless, the absence of other clinical more typical seizure manifestations, unremarkable neurological examination, and personal history of epilepsy make this diagnosis less likely.

Hallucinations are listed as primary diagnostic criteria for various psychotic disorders [15]. Insomnia, which is very common in people experiencing psychosis [16], was our patient's first symptom. However, the patient's insight into the fictional nature of his hallucinations and the fact that the visual hallucinations of schizophrenia are rare without auditory hallucinations makes this diagnosis highly unlikely [17]. Besides, late-onset schizophrenia tends to be associated with complex systematized delusions [18].

Overall, antidepressant treatment is associated with an increased risk of subsequent mania, which might include visual hallucinations [19]. However, in this case, there was no evidence of inflated self-esteem or overtalkativeness and no flight of ideas or racing thoughts.

Many prescription drugs can cause confusional states [20], and antidepressants have been cited as inducing hallucinations rarely [21, 22]. A subsyndromal delirium has been suggested for patients who have an incomplete form of delirium (only visual hallucinations, e.g.) [23]. Considering there was no history of hallucinations prior to the use of trazodone, and the temporal relation between the start and end of the patient's trazodone treatment and the onset of visual hallucinations, a mechanism of pharmacological induction makes the most likely explanation of this case.

## 4. Treatment

Trazodone was stopped and the patient has been started on mirtazapine 15 mg, at bedtime.

## 5. Outcome and Follow-Up

The hallucinations cleared up upon discontinuation of the drug, within 48 hours. The patient was provided also with supportive psychotherapy and psychoeducation on sleep hygiene. The patient reported sleep improvement, and, after a one-week stay at psychiatry department, he was discharged.

The patient started doing his routine activities and hallucinations did not recur during a follow-up period of 2 years.

## 6. Discussion

Trazodone was first approved by the Food and Drug Administration in 1981, as an antidepressant. According to recognized experts in the field, prescribing low dose trazodone as a hypnotic drug is considered to be the most frequent off-label use of a drug in all of psychopharmacology [24].

Trazodone is a multifunctional drug, with dose-dependent functions with more than one therapeutic mechanism. At low doses (50–150 mg), it has hypnotic actions due to blockade of serotonin receptors, as well as H_1_ histamine receptors and *α*_1_ adrenergic receptors, and at higher doses (150–600 mg) recruits additional pharmacological actions through the blockade of the Serotonin Transporter (SERT), becoming a full antidepressant [24].

The pharmacological profile of trazodone on serotonin (5-HT) system is highly complex and his therapeutic edge over other antidepressants might be due to its simultaneous action on 5-HT_2A_ and 5-HT_2C_ receptors [25]. 5-HT_2A/2C_ blockade can raise the levels of several neurotransmitters, such as dopamine and norepinephrine, in the prefrontal cortex, enhancing antidepressant effects [26]. In fact, on animal studies, trazodone at 30, 40, and 50 mg/kg, by blocking 5-HT_2C_ receptors stimulated the nigrostriatal dopaminergic neurons [27]. Although lacking direct effect on dopaminergic receptors, this indirect pathway might be relevant, considering the fact that hallucinations might be understood as a phenomenological correlate of dopaminergic dysfunction in the brain [28].

Albeit less pronounced than tricyclic depressants, trazodone has still effects on cholinergic transmission, and so hallucinations might also result from a relatively reduced cholinergic activity, induced by trazodone. Other central nervous system effects such as confusion and decreased concentration were also observed in patients taking trazodone [29]. A recent meta-analysis that looked for associations between drugs with anticholinergic effects and cognitive impairment and falls showed that Olanzapine and trazodone were associated with increased odds and risk of falls, as well as cognitive impairment, although the latest was more modest [30].

As we have seen, trazodone has serotonergic properties and is comparable, at least to some degree, to traditional serotoninergic antidepressants. There are several reports on the emergence of psychotic symptoms, including only visual hallucinations, during treatment with serotoninergic antidepressants [31], such as Sertraline [32], Duloxetine [33], and Citalopram [34]. Visual hallucinations might also be part of the discontinuation syndrome that results from Paroxetine discontinuation [35]. The serotoninergic model of hallucinations is related to cortical 5-HT_2A_ receptor hyperactivation, which appears to be the main mechanism of LSD induced psychosis [36]. Interestingly, a long history of LSD abuse might predispose to the occurrence of LSD flashback syndrome (characterized by transient visual hallucinations), following initiation of antidepressant therapy with selective serotonin reuptake inhibitor agents [37]. The underlying mechanism might be the destruction of inhibitory serotonergic interneurons caused by exposure of LSD, or serotonin receptors remaining in a state of permanent upregulation following previous LSD use. As a result, an acute increase of synaptic serotonin, from the initiation of SSRI treatment, would result in a highly enhanced serotonin signal, which might lead to hallucinogenic effects [38]. Our patient had a history of alcohol abuse. Similarly to LSD, we speculate that heavy alcohol use in the past may have induced similar changes in the brain, increasing vulnerability to the development of hallucinations, after antidepressant treatment (in this case, trazodone). Here we first report a case of visual hallucinations, induced by trazodone. Related findings were already observed and published in the literature, particularly auditory hallucinations induced by trazodone [39], and a disorganized type of psychosis in which the patient's psychotic symptoms included hallucinations as part of the clinical picture [40], after trazodone treatment. In all of them there was a clinical remission upon the discontinuation of the offending drug.

We would like to emphasize that trazodone-induced visual hallucinations, like any hallucinatory event, have the potential to be underreported. The low report of visual hallucinations may be explained by the embarrassment of the patient to report visions that they feel will not be perceived by others to be real, like in this case, where the patient was afraid to be confused by others as having a mental illness or excessive drug use.

In conclusion, a temporary dysfunction in neurotransmission involving serotoninergic and/or cholinergic pathways, induced by low dose trazodone administration, might contribute to the occurrence of hallucinations. Further studies or case reports are required regarding the underlying mechanisms of action before definitive conclusions can be reached.

## Additional Points

*Key Clinical Message*. (1) Visual Hallucinations are not always indicators of neurological or mental disorder. (2) A full drug history should be considered mandatory in any patient complaining of visual hallucinations. (3) Trazodone, like other antidepressants, might seldom precipitate hallucinations. The awareness of this rare mechanism avoids the burden of unnecessary antipsychotics.
